# Supplementary figures and images for: ApoE-Dependent Lipid Handling by Median Eminence Microglia Preserves Myelin Integrity and Metabolic Function
Source: bioRxiv. 2026 Feb 19:2026.02.18.706643. Preprint. [Version 1] doi: 10.64898/2026.02.18.706643 (PMC12934649; doi:10.64898/2026.02.18.706643)

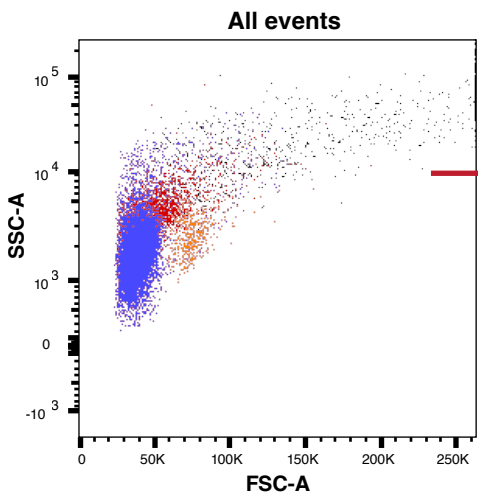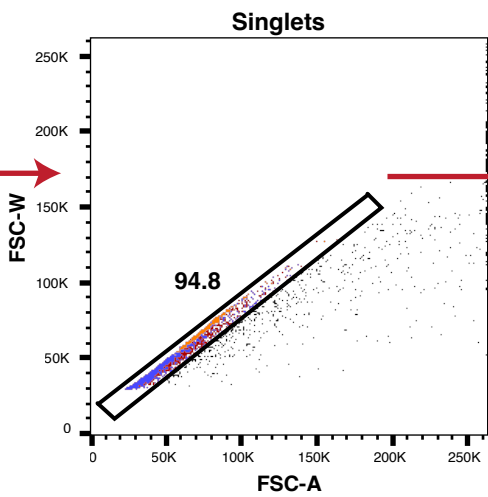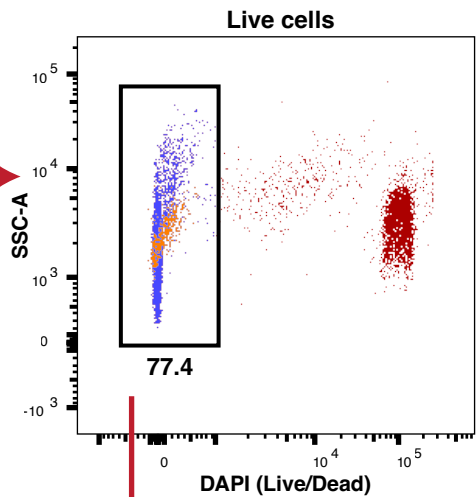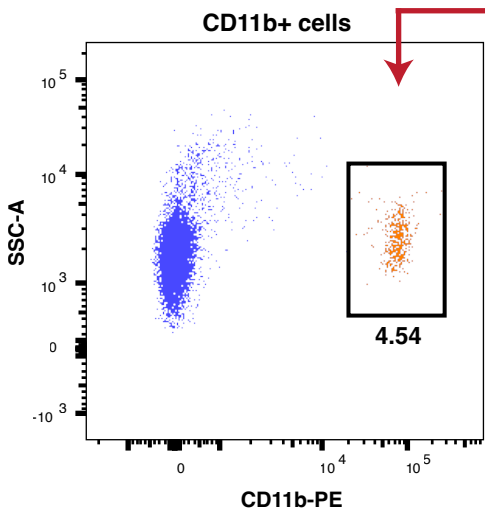

|   | Sample Name | Subset Name    | Count |
|---|-------------|----------------|-------|
| ■ | 36183.fcs   | CD11b+         | 448   |
| ■ | 36183.fcs   | Live (DAPI-)   | 9873  |
| ■ | 36183.fcs   | Singlets (FSC) | 12759 |
| ■ | 36183.fcs   | Ungated        | 13459 |

Supplement: Supplement 2 [file media-2.pdf]

**A**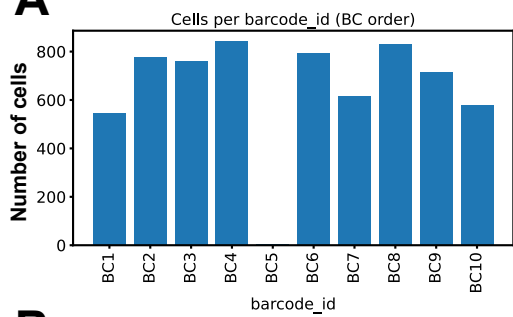**C**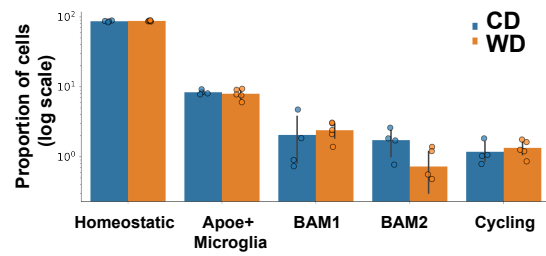**B**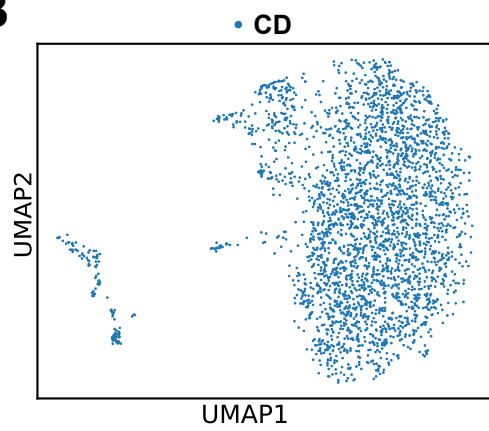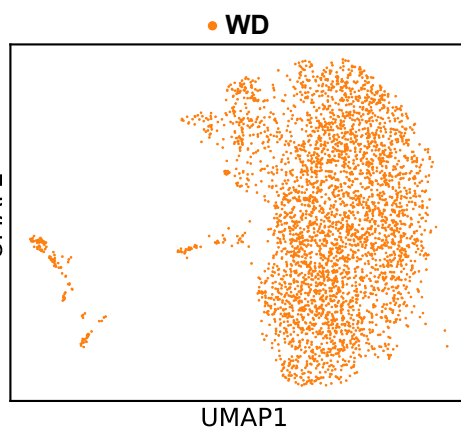

Supplement: Supplement 3 [file media-3.pdf]

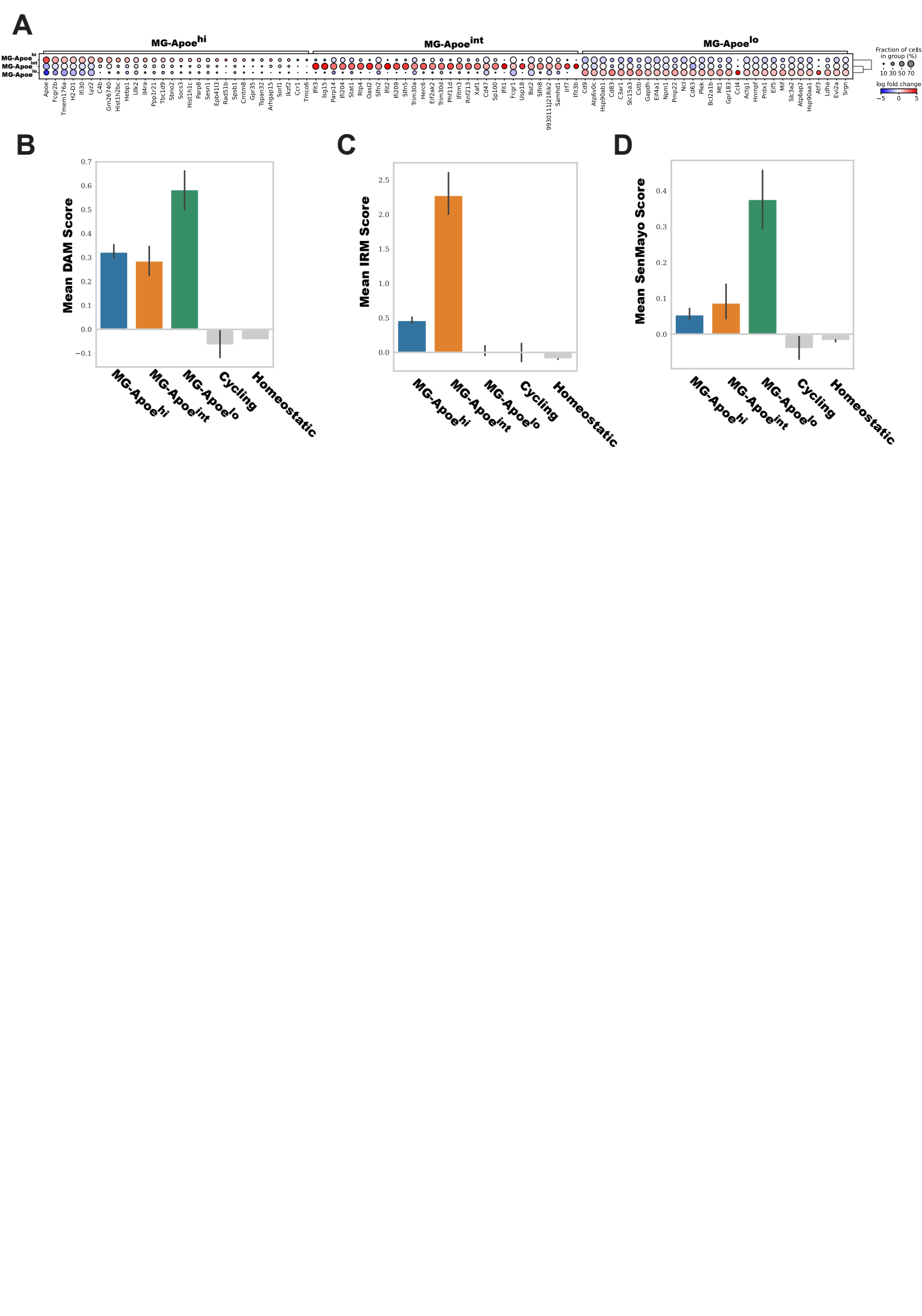

Supplement: Supplement 4 [file media-4.pdf]

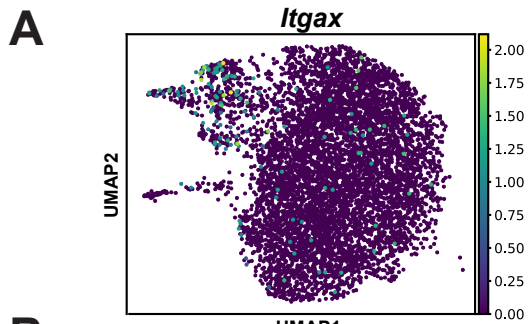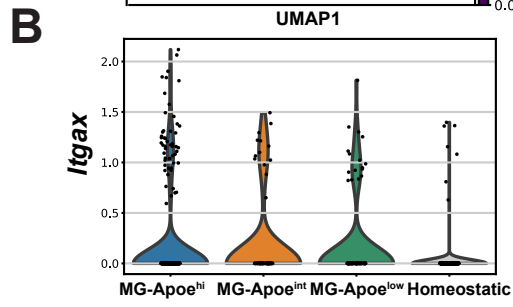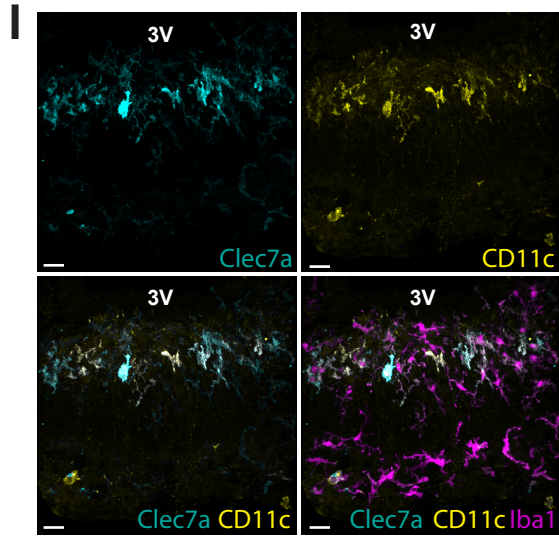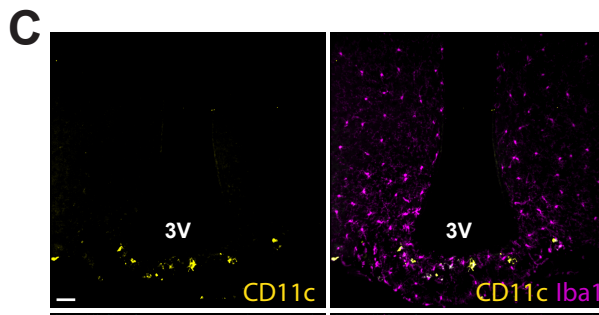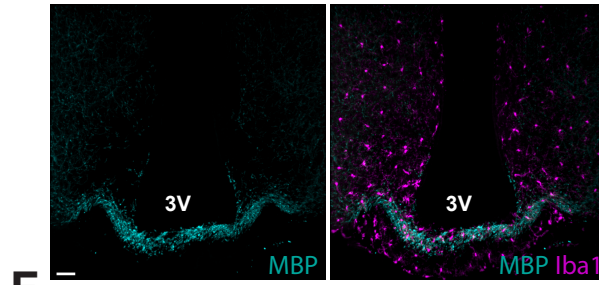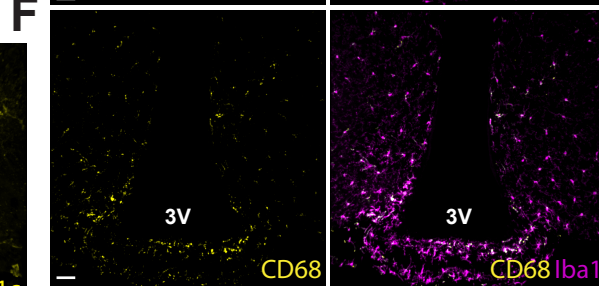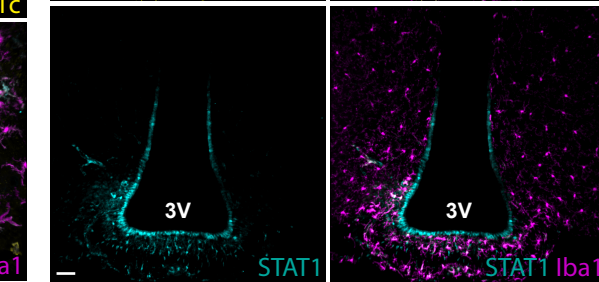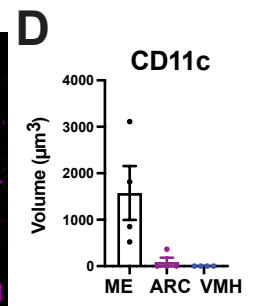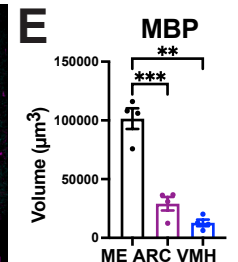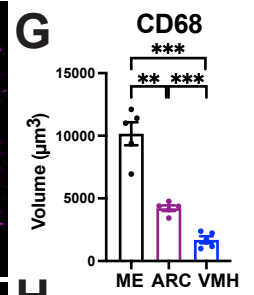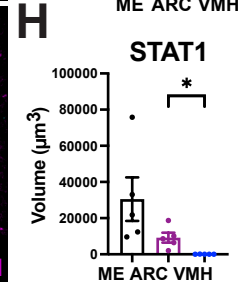

Supplement: Supplement 5 [file media-5.pdf]

**A**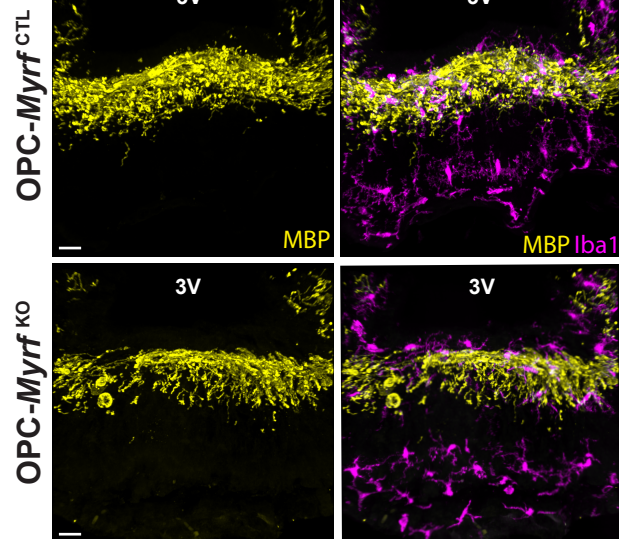**B****Myelin Basic Protein**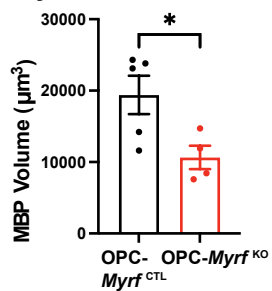**C****Microglial Myelin Processing**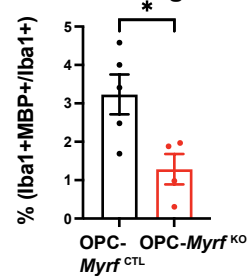**D**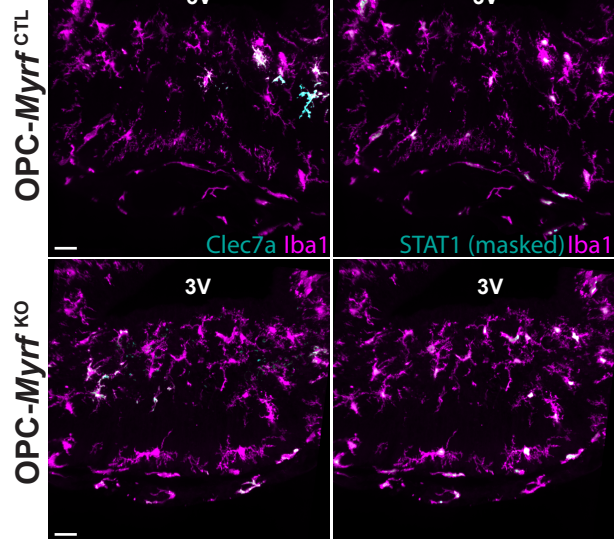**E****Clec7a<sup>+</sup> Microglia**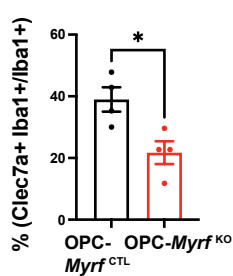**F****IRM Cells**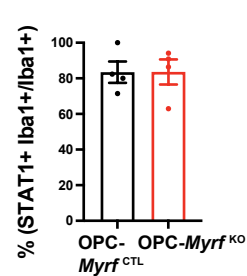

Supplement: Supplement 6 [file media-6.pdf]

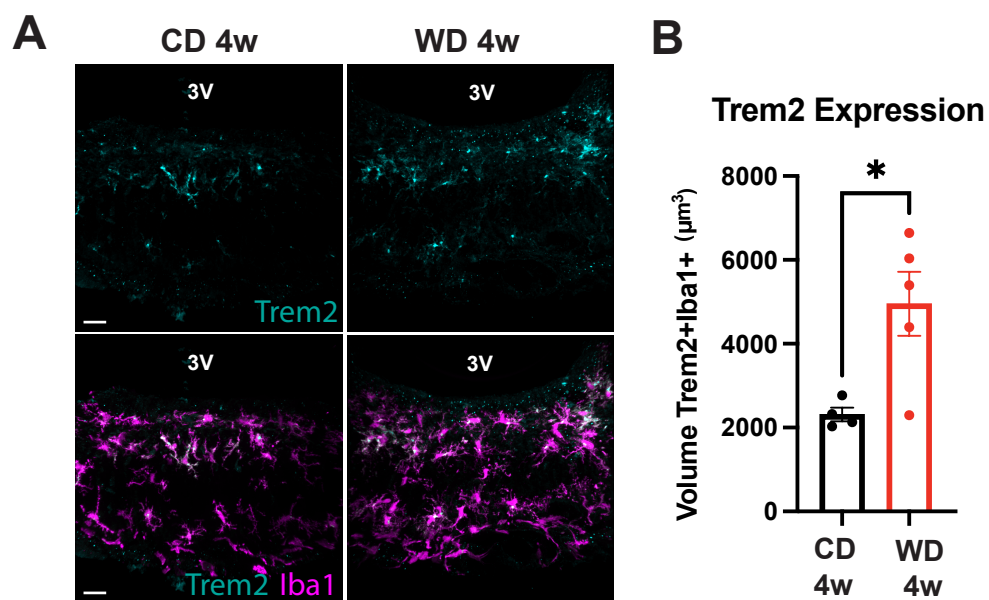

Supplement: Supplement 7 [file media-7.pdf]

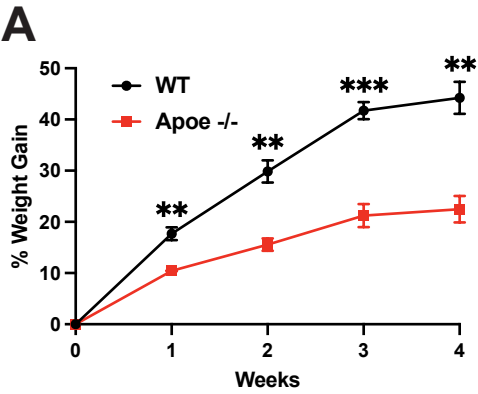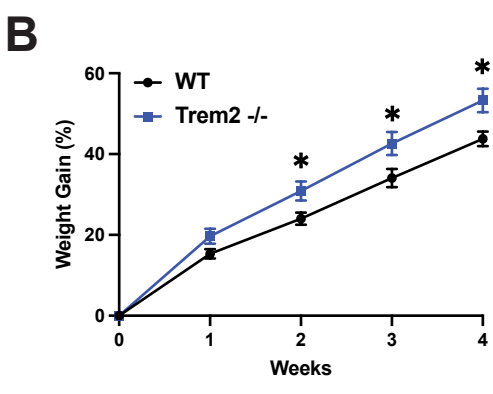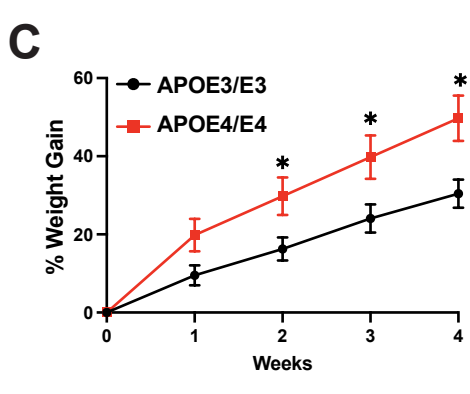

Supplement: Supplement 8 [file media-8.pdf]

**A**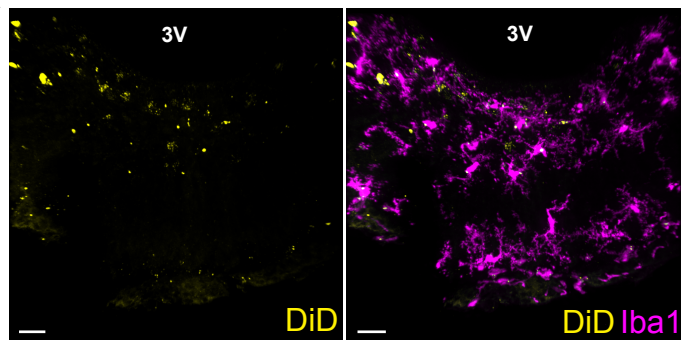**B**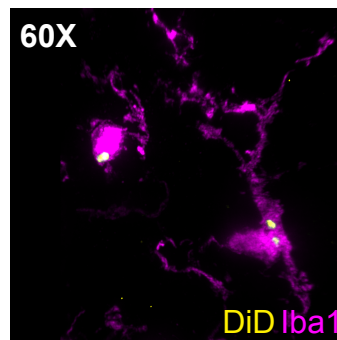**C**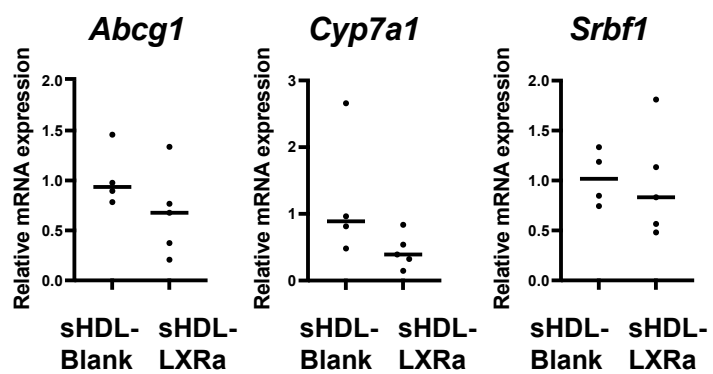**D**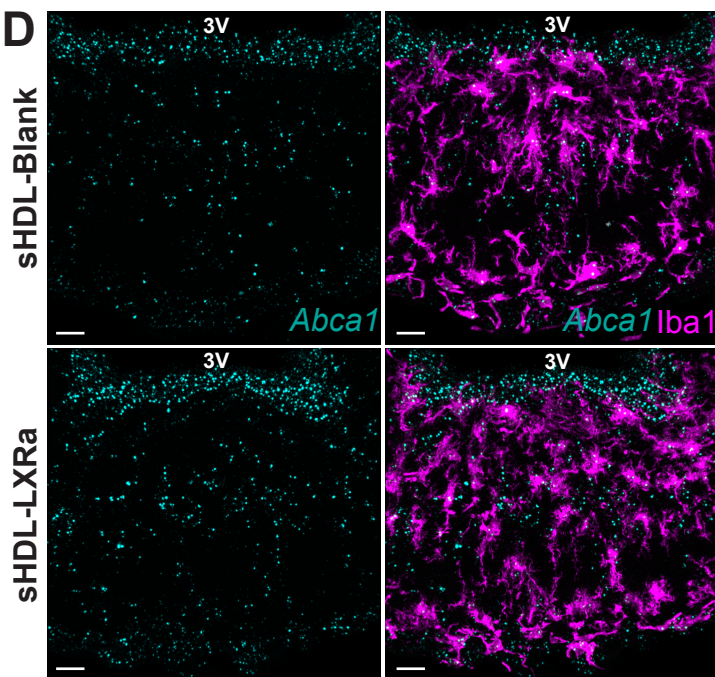**E**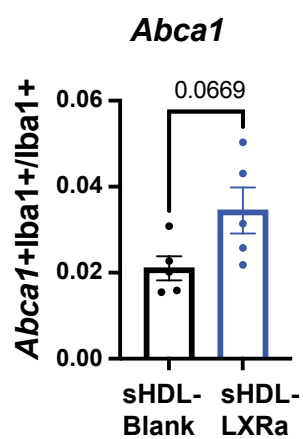

Supplement: Supplement 9 [file media-9.pdf]
